# Multimodal Therapy Approaches for NUT Carcinoma by Dual Combination of Oncolytic Virus *Talimogene Laherparepvec* with Small Molecule Inhibitors

**DOI:** 10.3390/v16050775

**Published:** 2024-05-14

**Authors:** Stavros Sotiriadis, Julia Beil, Susanne Berchtold, Irina Smirnow, Andrea Schenk, Ulrich M. Lauer

**Affiliations:** 1 Department of Medical Oncology and Pneumology, Virotherapy Center Tübingen (VCT), Medical University Hospital, 72076 Tübingen, Germany; stavros.sotiriadis@med.uni-tuebingen.de (S.S.);; 2 German Cancer Consortium (DKTK), Partner Site Tübingen, a Partnership between DKFZ and University Hospital Tübingen, 72076 Tübingen, Germany

**Keywords:** NUT carcinoma, talimogene laherparepvec, T-VEC, virotherapy, small molecule inhibitors, combination therapy, Myc

## Abstract

NUT (nuclear-protein-in-testis) carcinoma (NC) is a highly aggressive tumor disease. Given that current treatment regimens offer a median survival of six months only, it is likely that this type of tumor requires an extended multimodal treatment approach to improve prognosis. In an earlier case report, we could show that an oncolytic herpes simplex virus (T-VEC) is functional in NC patients. To identify further combination partners for T-VEC, we have investigated the anti-tumoral effects of T-VEC and five different small molecule inhibitors (SMIs) alone and in combination in human NC cell lines. Dual combinations were found to result in higher rates of tumor cell reductions when compared to the respective monotherapy as demonstrated by viability assays and real-time tumor cell growth monitoring. Interestingly, we found that the combination of T-VEC with SMIs resulted in both stronger and earlier reductions in the expression of c-Myc, a main driver of NC cell proliferation, when compared to T-VEC monotherapy. These results indicate the great potential of combinatorial therapies using oncolytic viruses and SMIs to control the highly aggressive behavior of NC cancers and probably will pave the way for innovative multimodal clinical studies in the near future.

## 1. Introduction

NUT carcinoma (NC), formerly known as NUT midline carcinoma (NMC), is a very rare cancer type causing very aggressive and highly resistant tumors that can arise anywhere in the body, but often appear along midline structures (head, neck, mediastinum) [1,2]. NC is well characterized by one single genetic translocation involving the NUT midline carcinoma family member 1 (*NUTM1*) gene, formerly known as the nuclear-protein-in-testis (*NUT*) gene, on chromosome 15q13 [3,4]. *NUTM1* can undergo fusion with several partners, such in most cases with *BRD4* (on chromosome 19q13) [5,6], but also, e.g., *BRD3* (on chromosome 9q34) [7] or *NSD3* (on chromosome 8p11-12) [8].

Although the exact function of *NUTM1* is not yet completely known, it is associated with poorly differentiated epithelial carcinomas that commonly occur in young adults and show an aggressive growth dynamic especially when these cells carry the *BRD4-NUTM1* fusion gene [3,7]. BRD4 belongs to the bromodomain and extra-terminal domain (BET) family of proteins that bind to acetylated histone marks and operate as reader domains in recognition of the epigenetic code by co-directing gene transcription [9,10]. Aberrant BRD4-NUT fusion proteins recruit histone acetyltransferase p300, allowing local hyperacetylation of chromatin and formation of “oncogenic megadomains” that primarily activate proliferation-enhancing genes [11,12]. In NC, a hallmark megadomain involves the MYC locus, which is a main driver of NC cell proliferation and is well known to have an additional important role in cell differentiation and cell cycle control [13,14,15]. 

An exact incidence of NC cannot be stated because it remains a highly underdiagnosed malignancy without morphological features that are often misdiagnosed with other tumor types (e.g., lymphomas, metastatic neuroendocrine tumors and sarcomas [16]), leading to untargeted treatments [17]. Current treatment approaches, which mostly rely on surgical resection, radiation and polychemotherapy, provide a median survival time of only about six months for the mostly affected adolescents and young patients [18,19,20]. Recent approaches, including standard chemotherapeutic regimens and first-generation BET inhibitors (BETi), have also been limited by low response rates, the development of severe resistances and the lack of significant efficacy [21,22,23]. These results indicate that this tumor type urgently requires an extended combination of multimodal and novel therapeutic approaches due to its highly aggressive behavior and pattern of early therapeutic resistance.

One of these novel approaches involves oncolytic virotherapy, whose efficacy in NC cell lines has recently been demonstrated in vitro [24]. Based on this, a recent case report demonstrates for the first time the feasibility of an innovative immunovirotherapy regimen in NC patients as an add-on to standard therapy with a profound and durable replication of T-VEC, which contributes significantly to tumor stabilization and improvement of the patient’s quality of life [25]. Oncolytic viruses (OVs) are designed as replication-competent vectors with the ability to infect tumor cells selectively. Dysregulated signaling pathways, like the inactivation of tumor suppressors or activation of oncogenes, not only provide an advantage to tumor cells but also promote viral replication, especially through the loss of antiviral defense mechanisms due to tumorigenesis [26,27]. This allows OVs to replicate massively, resulting in tumor cell lysis and the release of thousands of progeny viral particles. Once they infect neighboring tumor cells, they can subsequently spread throughout the whole tumor area and evoke an inflammatory environment that releases pathogen- and damage-associated molecular patterns (PAMPs and DAMPs), among others. This leads to an activation of the patient’s immune system, which is directed against the oncolytically released tumor-specific neo-antigens and triggers a profound systemic antitumor immune response [28]. 

In clinical translation, this mechanism could be exploited by using the proven efficacy of OVs to effectively target NC tumors that are resistant to conventional therapies [29]. This might be accomplished by *talimogene laherparepvec* (T-VEC), a genetically modified oncolytic herpes simplex virus type 1 (HSV-1) used in this study that has already been clinically approved in 2015 for the treatment of malignant melanoma. T-VEC incorporates two inserted copies of the immune-stimulating human granulocyte–monocyte colony-stimulating growth factor (GM-CSF) gene in its vector design [30,31]. 

However, a combination of oncolytic virotherapy with anti-proliferative compounds seems necessary to inhibit the mushrooming growth of NC cells by initially reducing the tumor cell mass until a sustained virotherapy-induced immune response can subsequently unfold. On this basis, we applied five distinct molecular compounds in this study, belonging to the category of *small molecule inhibitors* (SMIs), which have been shown to be able to reduce the viabilities of NC cells:

1. The pan-histone deacetylase inhibitors (HDACi) vorinostat and panobinostat counteract global hypoacetylation and suppression of transcribed genes required for cellular differentiation [32,33,34];
2. Fimepinostat as a dual-combined HDACi and phospho-inositol-3-kinase (PI3K) inhibitor exhibits a synergistic potential to downregulate Myc and induce apoptosis [35];
3. The selective p300/CBP bromodomain inhibitor GNE-781 inhibits p300-mediated histone acetylation in NC cells and thereby can prevent NUT fusion proteins from binding to accessible megadomains [36,37];
4. The cyclin-dependent kinase (CDK)-4/6 inhibitor (CDK4/6i) palbociclib prevents phosphorylation of the retinoblastoma (Rb) protein and disrupts the cell cycle in the CDK-mediated transition from G1 to S phase. Furthermore, palbociclib can also critically modulate the resistance of NC cells to BETi [38]. In addition, CDK4/6i was recently found to enhance the efficacy of an oncolytic adenovirus by modulation of the Rb protein [39].

This panel of SMIs may highlight excellent combination partners for multimodal approaches due to their favorable toxicity profiles and Myc-linked downstream target of BRD-NUT fusion proteins [32,40,41]. The aim of this study was to investigate whether the targetable impact of SMIs can support a T-VEC-based immunovirotherapy, providing a new set of options in multimodal treatment regimens. 

Here, we systematically examined the dual combination treatment of T-VEC with SMIs in five human NC cell lines, all carrying the aberrant *BRD4-NUT* fusion gene, with regard to efficacy and potential enhancement of anti-tumoral effects. In view of a future clinical application, we have also carried out tests to ensure that the two treatment regimens do not negatively affect each other. For this purpose, assays were performed to investigate cytotoxicity, real-time tumor cell proliferation, viral replication, as well as expression of the c-Myc protein (hereafter referred to as Myc) under dual combination therapy settings, which may reveal additional anti-tumoral effects for future clinical implementation.

## 2. Materials and Methods

### 2.1. NC Tumor Cell Lines

A panel of five human NUT carcinoma cell lines, all carrying the *BRD4-NUTM1* fusion gene, was used in this study. NC cell lines 690100, 143100 and HCC2429 were kindly supplied by Prof. Jens Siveke’s group at University Hospital Essen, Germany. NC cell lines 14169 and 10-15 were kindly provided by Prof. Christopher A. French, Boston, USA. All NC cell lines were authenticated by STR profiling at the German Collection of Microorganisms and Cell Cultures (DSMZ) in Braunschweig, Germany. Vero cells (African green monkey kidney cells) were purchased from DSMZ (No.: ACC 33) and were used for virus titrations only. All NC cell lines were cultured in DMEM supplemented with 10% FCS. Potential contamination with mycoplasma was tested negatively for all cell lines before use by a PCR-based MycoSPY kit system (Biontex, Munich, Germany).

### 2.2. Treatment with Small Molecule Inhibitors (SMIs)

Five SMIs, i.e., vorinostat (suberoylanilide hydroxamic acid, SAHA), panobinostat (LBH589), fimepinostat (CUDC-907), GNE-781, and palbociclib (PD-0332991) were purchased from Selleckchem (Cologne, Germany). The medium was replaced 24 h after seeding of the NC tumor cells in 24-well plates with a fresh medium containing the appropriate treatment concentration of the respective SMIs and NC cells were incubated until the selected endpoints.

### 2.3. Virus Infections

The OV T-VEC, a modified herpes simplex virus type 1 (HSV-1), was kindly provided by Amgen Inc. (Thousand Oaks, CA, USA). NC cells were infected with T-VEC as previously described [42]. Only serum-free medium was used in MOCK treatments. In the combinatorial treatment regimens, the medium was replaced one hour post-infection (hpi) with a medium containing SMIs in their respective concentrations.

### 2.4. Sulforhodamine B Cell Viability Assay

The viability of the NC cell lines was determined in 24-well-plates at 96 h post-treatment (hpt) after monotherapy with SMIs and at 72 hpi with T-VEC alone or in the dual combination regimens employing a distinct SMI using the Sulforhodamine B (SRB) cell viability assay, as described previously [42].

### 2.5. Virus Growth Curves

NC cells were seeded in 6-well plates and infected with T-VEC at the specified multiplicities of infection (MOIs) with or without the addition of a distinct SMI. Viral replication was quantified by performing plaque assays at 1, 24, 48, 72 hpi, as described previously [42].

### 2.6. Real-Time Cell Proliferation Assays

NC cells were seeded in E-96-well plates and infected 24 h later with T-VEC. At 1 hpi distinct SMIs were added. Real-time dynamic cell proliferation was measured every 30 min during the full observation time of 96 h by xCELLigence RTCA SP System (Roche Applied Science, Penzberg, Germany). The cell impedance, i.e., the electrical resistance to alternating current, was expressed in an unspecified unit as the cell index. Cell index values were calculated using the RTCA software (1.2.1; Roche Applied Science, Penzberg, Germany). 

### 2.7. Western Blots

To prepare cell lysates, NC cells were seeded in 6-well plates and infected 24 h later with T-VEC in 1 mL DMEM using selected MOIs. For dual combination treatment, the medium was replaced at 1 hpi with 2 mL DMEM supplemented with 10% FCS containing SMIs at the appropriate concentrations. After incubation for 24, 48, and 72 hpi, wells were scraped off. After a first centrifugation step, then resuspension in PBS and another centrifugation step, the pellet was resuspended in 200 µL lysis buffer (containing 50 mM Tris pH 7.6, 150 mM NaCl, 1% IGEPAL, complete proteinase inhibitor (Roche)) and placed on ice. After three freeze–thaw cycles, samples were centrifuged at 4 °C for 10 min at 13,000 rounds per minute (rpm). Protein concentration in the supernatants was determined by Bradford assay (Bio-Rad, Hercules, CA, USA). An amount of 50 µg protein was diluted in eightfold SDS gel-loading buffer (modified from [43], added with 8% 2-mercaptoethanol) and denatured at 95 °C for 5 min. Proteins were separated by 8% SDS-PAGE (modified from [44]) and blotted onto a hydrophobic polyvinylidene difluoride (PVDF) membrane (Hybond-P, GE Healthcare, Waukesha, WI, USA).

After blocking with 5% nonfat dry milk (Carl Roth) in Tris-buffered saline (TBS) containing 0.02% Tween-20 (TBS-T), membranes were incubated with primary antibodies overnight at 4 °C (anti-c-Myc, 1:500, Cell Signaling Technology Inc. (Danvers, MA, USA); anti-Vinculin (1:5000), anti-ß-Actin (1:4000), Sigma-Aldrich Inc. (Saint Louis, MO, USA)). After washing the membranes three times with TBS-T, the secondary antibody (horseradish peroxidase-conjugated anti-rabbit or anti-mouse (Bio-Rad, 1:8000) in blocking buffer) was added for 1 h and membranes were washed three times with TBS-T. Proteins were detected with Amersham ECL Western Blotting Detection reagents (GE Healthcare). Molecular weight was determined using a pre-stained protein ladder (PagerRuler Plus, Thermo-Scientific, Waltham, MA, USA). Image acquisition and densitometric analysis of the bands were performed in the ChemiDoc MP Imaging System using Image Lab software version 5.1 (Bio-Rad Laboratories Inc., Hercules, CA, USA).

### 2.8. Statistical Analysis

Statistical analysis was performed using GraphPad Prism version 9 (GraphPad Software Inc., San Diego, CA, USA). The reductions in NC cell viability, comparisons of viral growth and anti-proliferative effects between two treatment groups in vitro were analyzed using Brown–Forsythe and Welch ANOVA and Dunnett’s multiple comparison test. Welch’s *t*-test was used in cases where one experiment was performed. Four different *p* values were determined: *p* < 0.05 (*), *p* < 0.01 (**), *p* < 0.001 (***), *p* < 0.0001 (****).

## 3. Results

### 3.1. Dual Combination Therapies (T-VEC with SMIs) Reduce Viability of NC Cancer Cell Lines

In order to determine possible anti-tumoral effects in vitro, monotherapeutic approaches with T-VEC or a singular SMI (vorinostat, panobinostat, fimepinostat, GNE-781, or palbociclib) were evaluated in a first step. For this purpose, all five NC cell lines were first mono-treated with a respective SMI in graded concentrations and the remaining NC tumor cells were determined after 96 h by SRB viability assays. 

A concentration-dependent reduction in NC tumor cells was observed in all five NC cell lines after monotherapy with vorinostat, panobinostat, fimepinostat, GNE-781 and palbociclib, requiring only very low nanomolar to micromolar ranges of the respective compounds (Supplementary Figures S1–S5). This was found to be consistent with earlier findings [32,37]. 

An overall high susceptibility was found in HCC2429 cells, particularly when being treated with panobinostat and fimepinostat at low nanomolecular dosages (0.5 to 25 nM; Supplementary Figure S1B,C). In NC cell line 14169, the remaining tumor cell viability initially increased slightly under treatment with low concentrations of all SMIs, except for GNE-781. When higher concentrations of the respective SMIs were applied, a gradual reduction in the NC tumor cell viability was observed (Supplementary Figure S5). 

For T-VEC monotherapy, the viability of NC cells was assessed analogously for all five NC cell lines by infecting them in graded MOIs (ranging from 0.00001 up to 0.01) or leaving them uninfected (MOCK). At 72 hpi, increasing MOIs went along with strikingly decreased NC tumor cells, even under very low MOIs as being the case with NC cell lines 143100, 10-15 and 14169 (Supplementary Figure S6). The displayed significance levels were determined for the lowest MOI that caused a significant result, respectively, indicating highly significant NC tumor cell reductions (when compared to MOCK treatments) in 143100, 10-15 and 14169 cells under extremely low MOIs (0.00025; 0.0005; 0.00005). The occasionally high standard deviations for T-VEC can be explained by the very low applied MOI levels and the associated variations. 

**Figure 1:**
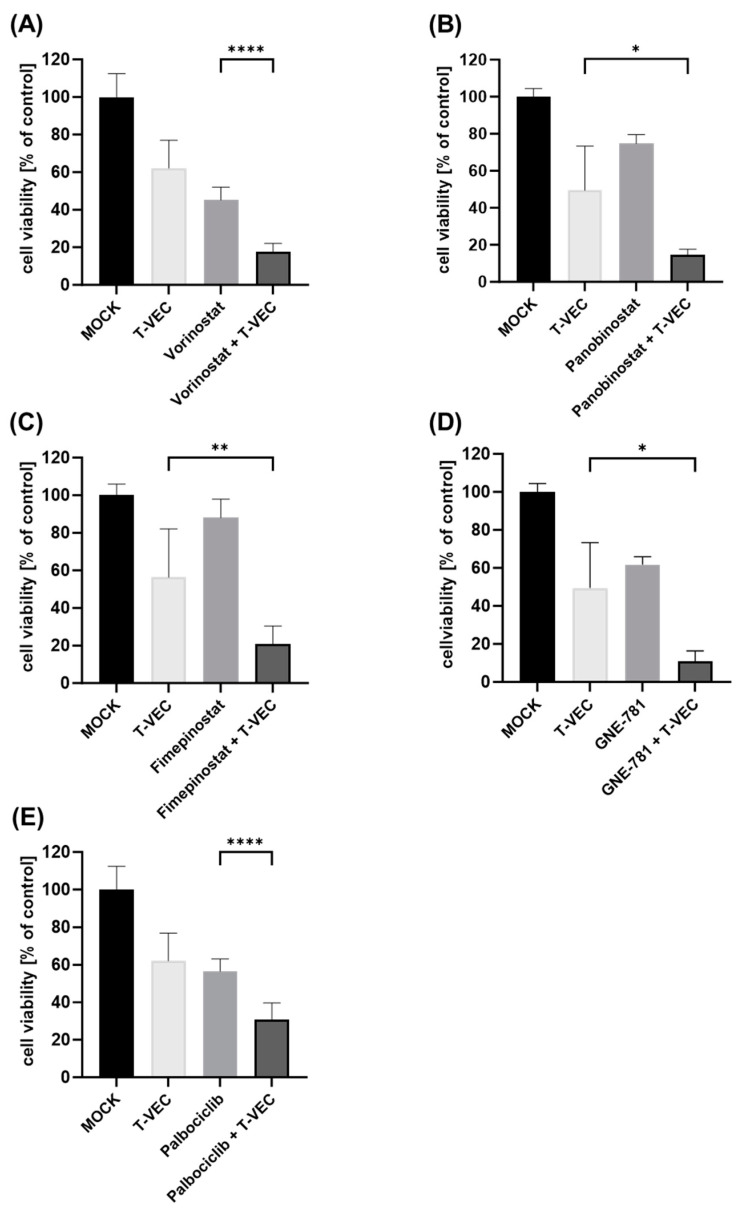
Viability of human NC cell line HCC2429 after dual combination treatment with T-VEC and small molecule inhibitors (SMIs): HCC2429 cells were infected with T-VEC at a multiplicity of infection (MOI) of 0.001 and treated with the SMIs vorinostat (500 nM) (**A**), panobinostat (2.5 nM) (**B**), fimepinostat (0.5 nM) (**C**), GNE-781 (25 nM) (**D**), and palbociclib (100 nM) (**E**) in dual combination regimes or alone, or remained untreated (MOCK). The remaining NC tumor cells were determined at 72 h post-infection (hpi) by SRB viability assays. The mean ± SD of at least two independent experiments performed in quadruplicates is shown. Reported significances refer to dual combination compared to the respective monotherapy with the lowest remaining cell viability. * *p* < 0.05; ** *p* < 0.01, **** *p* < 0.0001.

**Figure 2:**
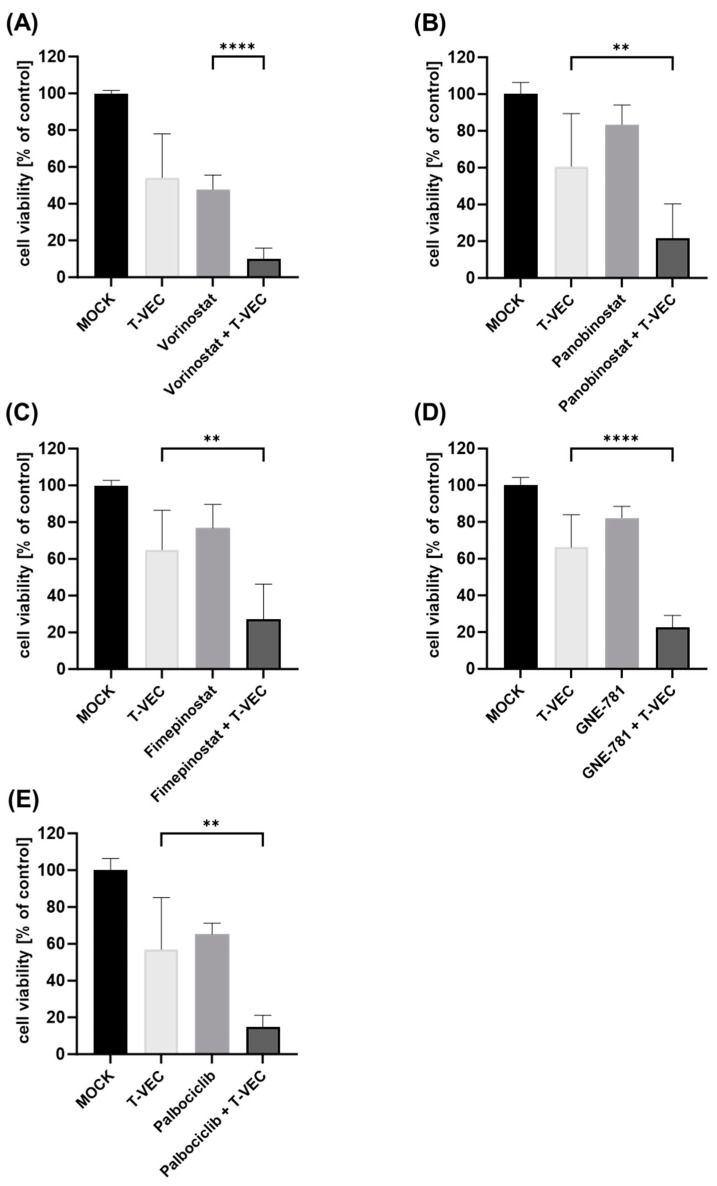
Viability of human NC cell line 143100 after dual combination treatment with T-VEC and small molecule inhibitors (SMIs): 143100 cells were infected with T-VEC at a multiplicity of infection (MOI) of 0.00005 and treated with the SMIs vorinostat (1 µM) (**A**), panobinostat (2.5 nM) (**B**), fimepinostat (1 nM) (**C**), GNE-781 (25 nM) (**D**), and palbociclib (100 nM) (**E**) in dual combination or alone, or remained untreated (MOCK). The remaining NC tumor cells were determined at 72 h post-infection (hpi) by SRB viability assays. The mean ± SD of at least two independent experiments performed in triplicates is shown. Reported significances refer to dual combination compared to the respective monotherapy with the lowest remaining cell viability. ** *p* < 0.01, **** *p* < 0.0001.

**Figure 3:**
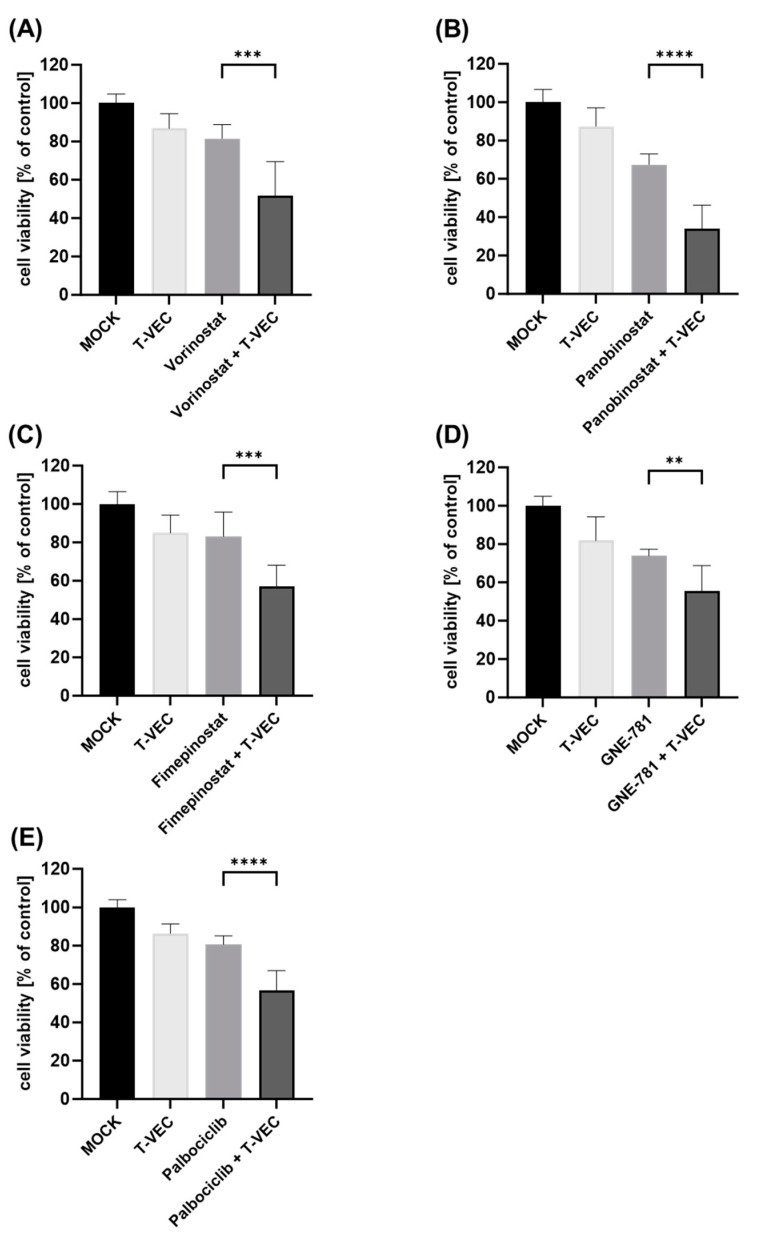
Viability of human NC cell line 10-15 after dual combination treatment with T-VEC and small molecule inhibitors (SMIs): 10-15 cells were infected with T-VEC at a multiplicity of infection (MOI) of 0.0005 and treated with the SMIs vorinostat (500 nM) (**A**), panobinostat (2.5 nM) (**B**), fimepinostat (0.5 nM) (**C**), GNE-781 (10 nM) (**D**), and palbociclib (25 nM) (**E**) in dual combination regimes or alone, or remained untreated (MOCK). The remaining NC tumor cells were determined at 72 h post-infection (hpi) by SRB viability assays. The mean ± SD of at least two independent experiments performed in quadruplicates is shown. Reported significances refer to dual combination compared to the respective monotherapy with the lowest remaining cell viability. ** *p* < 0.01, *** *p* < 0.001, **** *p* < 0.0001.

In a second step, the viability of the five NC cell lines after dual combination therapy (T-VEC with a distinct SMI, respectively) was investigated using SRB cell viability assays. In order to draw conclusions about additional effects when employing dual combination regimens, SMI concentrations as well as T-VEC MOI levels were selected that reduced NC tumor cells to approximately 60% in monotherapeutic applications. Furthermore, a treatment duration of 72 hpi was set for these regimens, since monotherapy with T-VEC over 96 hpi has already shown almost complete oncolysis of NC cells in our own prior experiments [24]. The NC cell lines HCC2429 (Figure 1), 143100 (Figure 2) and 10-15 (Figure 3) exhibited enhanced anti-tumoral effects with each and every dual combination regime when compared to the most potent monotherapy (*p* < 0.05 to *p* < 0.0001). Among all five SMIs tested, vorinostat and palbociclib showed the best results. Monotherapy with SMIs was able to decrease the viability of HCC2429 cells (Figure 1) by vorinostat (to 45%) and palbociclib (to 57%) compared to untreated cells, while the combinatorial treatments resulted in even higher reductions (to 18% by vorinostat + T-VEC; to 31% by palbociclib + T-VEC). 143100 cells (Figure 2) were susceptible to T-VEC under a very low MOI (0.00005) and the most potent monotherapy with vorinostat was able to halve the amount of NC cells (to 48%), compared to untreated cells. Furthermore, a dual combination enabled an additional significant reduction to 10% by vorinostat + T-VEC, compared to untreated cells. It was also remarkable that GNE-781 + T-VEC demonstrated a highly significant reduction of 143100 cells to 22% compared to untreated cells, while T-VEC alone led to a reduction to 66%. In 10-15 cells (Figure 3), the use of the HDACi compound panobinostat was slightly more effective than the application of the HDACi compound vorinostat. Although panobinostat alone decreased NC cells to 68%, panobinostat + T-VEC caused a highly significant reduction to 34% in dual combination when compared to untreated cells.

Similar findings could be observed in the other two NC cell lines (690100, 14169) as well (Supplementary Figures S7 and S8), although panobinostat in NC cell line 690100 and both vorinostat and palbociclib in NC cell line 14169 showed no further notable reductions in NC tumor cells in combinatorial approaches.

### 3.2. Dual Combination Therapies (T-VEC with SMIs) Inhhibit NC Cell Proliferation More Effectively

To further support the statement of enhanced cancer cell killing by dual combination therapies, real-time proliferation studies were performed in all five NC cell lines over 96 h, which included the treatment with T-VEC and one of the five SMIs at NC cell line-adjusted concentrations alone or in dual combination. 

**Figure 4:**
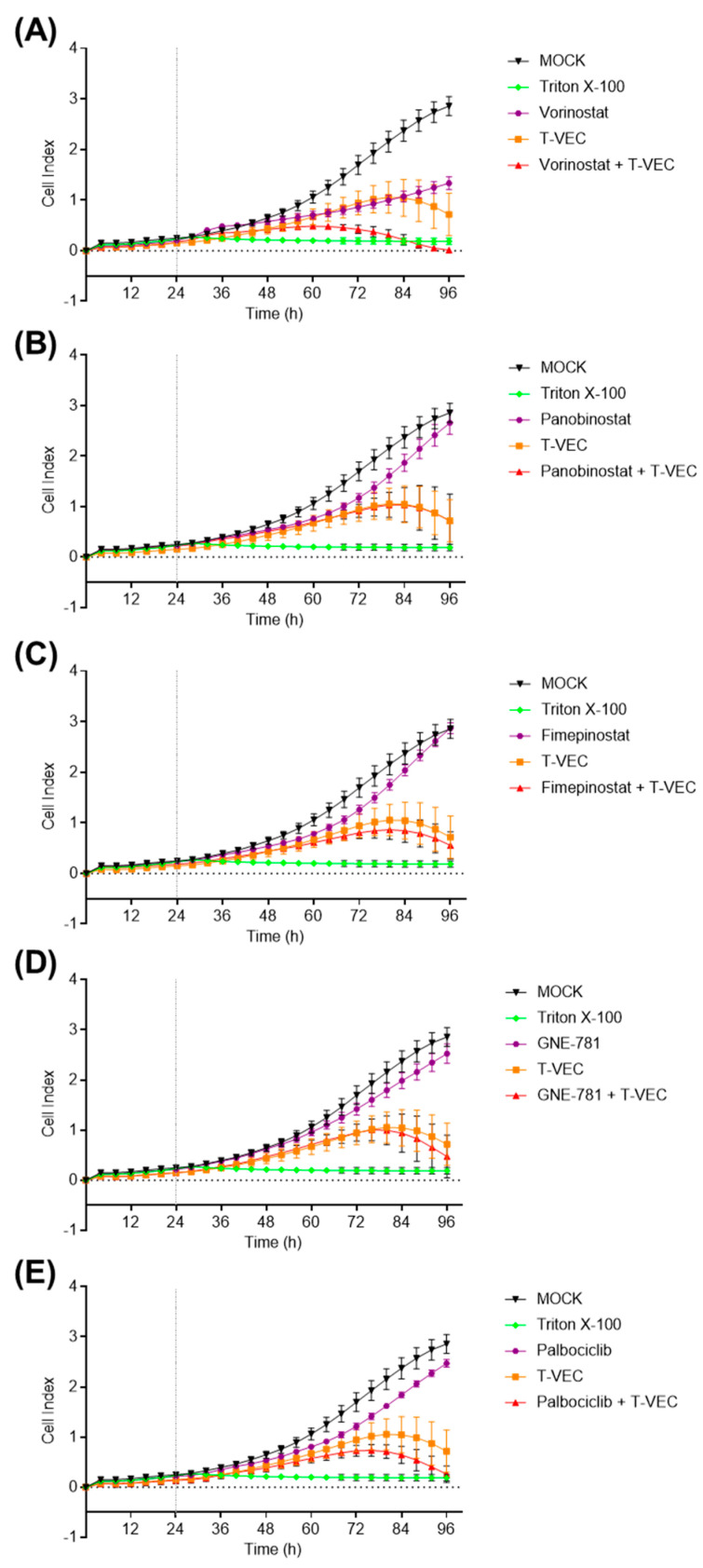
Real-time analysis of HCC2429 NC tumor cells under treatment with T-VEC alone or under dual combination therapies with small molecule inhibitors (SMIs). HCC2429 tumor cells were treated at 24 h after seeding with T-VEC (MOI 0.001) or with the SMI at the indicated concentration alone (vorinostat (500 nM) (**A**), panobinostat (2.5 nM) (**B**), fimepinostat (0.5 nM) (**C**), GNE-781 (25 nM) (**D**), and palbociclib (100 nM) (**E**)) or in combination. Triton X-100 0.1% was added as a negative control, resulting in maximal tumor cell lysis. Real-time cell proliferation was monitored using the xCELLigence^®^ RTCA SP system 1.2.1. Cell index on the y-axis is defined by measured electrode impedance. Vertical dashed lines mark the point of infection. MOCK, Triton X-100 and T-VEC curves are the same in each graph. Mean ± SD of one representative experiment at least performed in quadruplicates is shown.

In NC cell line HCC2429, it was exemplified that mono-treatment with all SMIs resulted in reduced cell proliferation (Figure 4). For example, vorinostat was able to achieve an impressive deceleration of the proliferation curve (Figure 4A). When infected with T-VEC alone, HCC2429 cells initially exhibited a flattened cell index. However, at around 60 hpi, cell proliferation began to decrease. Combinatorial approaches resulted in slightly stronger and earlier declined HCC2429 cell proliferation patterns when compared to T-VEC monotherapy, except for panobinostat + T-VEC (Figure 4B).

Taken together, all five NC tumor cell lines, when treated with dual combination regimes, were found to exhibit cell indices that flattened earlier and showed decreasing trends (Figure 4 and Supplementary Figures S9–S12), when compared to the monotherapeutic regimen. Moreover, in 14169 cells (Supplementary Figure S12), it was noticed that vorinostat, panobinostat, fimepinostat, and GNE-781 initially increased the cell index in monotherapy settings, but then exhibited flattened patterns over time. However, their combination with T-VEC allowed the curve to fall earlier and more steeply than after treatment with T-VEC alone. 

### 3.3. Dual Combination Therapies (T-VEC with SMIs) Promote Suppression of Myc Protein Expression

To investigate a molecular correlate of dual combination regimens by T-VEC with SMIs, immunoblots determining Myc protein expression were performed. For this purpose, NC cell line HCC2429 was exemplarily used as a model to detect possible regulations of Myc under both mono-treatment with T-VEC (MOI 0.001) or distinct SMIs as well as under combinatorial approaches (T-VEC plus SMIs) at the concentrations shown above. Expression of housekeeping genes ß-Actin and Vinculin was used as the loading control.

**Figure 5:**
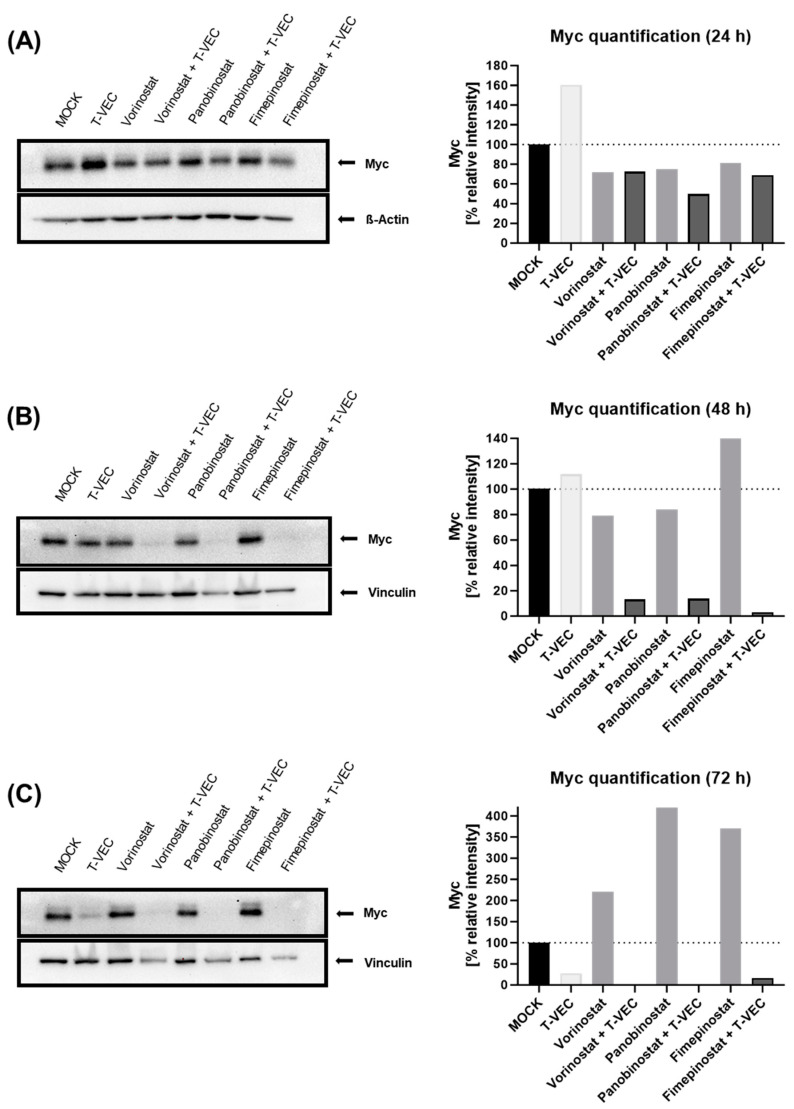
Modulation of c-Myc (Myc) protein expression after treatment of HCC2429 cells for 24 (**A**), 48 (**B**) and 72 (**C**) hours (h) with T-VEC and small molecule inhibitors (SMIs) alone or in dual combination. HCC2429 cells were treated with T-VEC (MOI 0.001), vorinostat (500 nM), panobinostat (2.5 nM), fimepinostat (0.5 nM) alone or T-VEC in dual combination with a distinct SMI or remained untreated (MOCK). Anti-ß-Actin and anti-Vinculin antibodies were used to provide loading controls. Quantification of Myc band intensities as a function of the control bands (ß-Actin, Vinculin) is shown in the graphs on the right at 24 hpi (**A**), 48 hpi (**B**) and 72 hpi (**C**), calculated relative to MOCK.

In order to possibly determine the timeline of the modulatory effects on Myc, protein expression of Myc after treatment with T-VEC and selected SMI compounds vorinostat, panobinostat, and fimepinostat as monotherapy and under dual combination was analyzed at 24 hpi (Figure 5A and Supplementary Figure S13), 48 hpi (Figure 5B and Supplementary Figure S14) and 72 hpi (Figure 5C and Supplementary Figure S15). 

It was apparent that Myc expression initially increased at 24 hpi under T-VEC mono-therapy relative to MOCK (Figure 5A), but then was increasingly suppressed over time (Figure 5B,C). Interestingly, dual combination treatments employing T-VEC with SMI compounds vorinostat, panobinostat, or fimepinostat reduced expression of Myc over the time starting already at 24 hpi (Figure 5A–C): At 24 hpi, Myc expression was more downregulated by panobinostat + T-VEC and fimepinostat + T-VEC compared with SMI monotherapy, and Myc expression was at nearly the same level by vorinostat with or without T-VEC. 

At 48 hpi, further significant modulatory effects on Myc were displayed under dual combination. Whereas vorinostat + T-VEC, panobinostat + T-VEC and fimepinostat + T-VEC were able to suppress Myc expression dramatically, monotherapy with T-VEC or SMIs did not change Myc levels significantly.

When compared with untreated NC cells (MOCK), T-VEC monotherapy led to a strong reduction in Myc expression at 72 hpi (Figure 5C). Of note, T-VEC in combination with SMI compounds vorinostat, panobinostat and fimepinostat resulted in almost complete suppression of Myc expression. Thus, the combination treatments demonstrated additive effects that could further downregulate Myc expression levels even below the values of T-VEC monotherapy.

### 3.4. Viral Replication of T-VEC Is Not Affected by SMIs in NC Tumor Cells

In the next step, viral titers of T-VEC were investigated for the respective observation time of 72 h with or without the presence of a distinct SMI compound besides T-VEC in all five NC cell lines. This examination can provide information on whether both therapies (OV and SMI) have a negative effect on each other, which could constitute an exclusion criterion for a possible combined therapeutic use in NC patients in the future. Once again, the cell-line-adjusted low MOI levels (0.00005 to 0.001) were employed in this setting.

**Figure 6:**
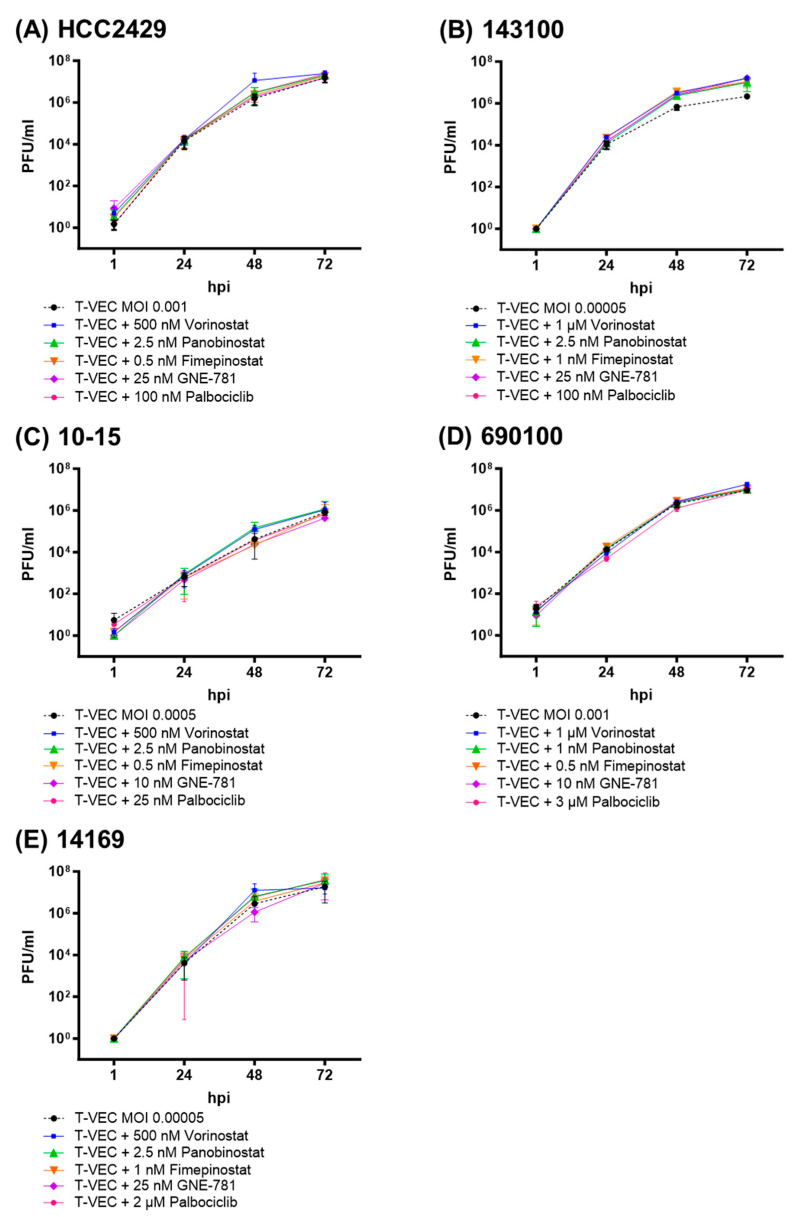
Replication of T-VEC in NC cell lines HCC2429 (**A**), 143100 (**B**), 10-15 (**C**), 690100 (**D**), 14169 (**E**) alone and after combination with small molecule inhibitors (SMIs): vorinostat, panobinostat, fimepinostat, GNE-781, or palbociclib was given at cell line-adjusted concentrations to NC tumor cells infected with T-VEC at cell line-adjusted multiplicity of infection (MOI). Viral replication was analyzed via plaque assay at 1, 24, 48 and 72 h post-infection (hpi). At least two independent experiments performed in duplicates are represented. PFU; plaque forming unit.

In general, T-VEC monotherapy resulted in maximum titer values between 10^6^ and 10^7^ plaque-forming units (PFU)/mL within 72 hpi for all five tested NC cell lines, indicating a high susceptibility of all five NC cell lines to T-VEC replication, which was found to be consistent with the results of prior experiments [24]. Replication of T-VEC was not negatively affected by any of the SMIs used in the combinatorial approaches as shown below for all five NC cell lines used (Figure 6).

In detail, viral titers of T-VEC showed a rapid increase in the NC cell line HCC2429 (Figure 6A) to 10^4^ PFU/mL within 24 hpi and a maximum increase to 10^7^ PFU/mL at 72 hpi, without reaching a plateau. Rather, a slight increase in replication was observed especially for T-VEC + vorinostat at 48 hpi with subsequent plateau settings, about one power of ten higher than achieved with T-VEC alone. For NC cell line 143100, it was observed that all dual combinations increased the viral titers by approximately one power of ten after 24 hpi compared to T-VEC alone, but did not reach a clear plateau after the maximum titer increase from 0 to 10^7^ PFU/mL when vorinostat, panobinostat, fimepinostat, GNE-781, or palbociclib were added (Figure 6B). 

As expected, all five NC cell lines allowed robust and high viral replication of T-VEC with viral titers up to 10^7^ PFU/mL within 72 hpi. No impairment in replication of T-VEC was observed in combination with the SMIs vorinostat, panobinostat, fimepinostat, GNE-781, or palbociclib. The increased level of viral replication observed by almost every dual combination treatment, while not statistically significant, correlated with decreasing NC tumor cell viability assays in the more susceptible 143100 cell line. An enhancement of replication of T-VEC in combination with vorinostat was observed in all NC cell lines. 

## 4. Discussion

To improve the prognosis of the highly challenging NC tumor entity, it not only seems to be necessary to further raise awareness for this rare cancer disease, but also to identify further suitable targeted therapies. Current standard approaches, based on surgical resections, chemoradiation and immune checkpoint inhibitor applications, often fail to achieve significant therapeutic outcomes [21].

To overcome this problem, immunovirotherapy with T-VEC could provide a novel therapeutic option that already has been demonstrated to be efficacious in NC cells both in vitro and in vivo [24,25]. Since monotherapy with BETi compounds has not shown any significant benefits in clinical trials [23], preclinical results from combinatorial treatments of BETi with T-VEC [24] and BETi with CDK4/6i [38] suggest possible significant improvements. The results of clinical trials, which investigate BETi compound ZEN-3694 in combination with CDK4/6i compound abemaciclib (NCT05372640) and the combination of BETi compound ZEN-3694 with chemotherapy, i.e., etoposide and cisplatin (NCT05019716) must be awaited. Of note, also a dual BET and p300/CBP inhibitor (EP31670) is currently in phase I testing (NCT05488548). 

In order to provide further targeted therapy options, here dual combination regimes of T-VEC with SMIs (vorinostat, panobinostat, fimepinostat, GNE-781, palbociclib) were investigated, demonstrating enhanced anti-tumoral responses in vitro and also providing first insights into anti-tumoral molecular mechanisms, such as modulation of Myc expression. 

Our data from SRB viability and proliferation assays correlate with each other and illustrate an additional anti-tumoral response induced by T-VEC with SMIs in dual combination compared to monotherapy. Since no impairment of T-VEC replication was observed in all five NC cell lines in the presence of SMIs and the more susceptible cell line 143100 even showed increased viral replication under dual combination treatment (Figure 6B), SMIs could be suitable candidates for joint use with virotherapeutics such as T-VEC in multimodal therapy regimens.

One difficulty was to find appropriate MOI levels for T-VEC in each NC cell line that still left space to reveal additional possible effects in dual combination settings. In particular, cell lines 143100 (Figure 3) and 14169 (Supplementary Figure S8) showed greater variations in the reduction in NC cell viability and thus larger standard deviations, which may be explained by the fact that very low MOI values (0.00005) were used for T-VEC. Fluctuations at 1 hpi in the replication curves of T-VEC with SMIs (Figure 6A,C) could also be attributed to a naturally fluctuating initial amount of added virus particles due to the very low MOI. Real-time cell monitoring also revealed that monotherapy with SMIs sometimes exceeded the measured cell index of MOCK-treated cells, which were more susceptible to T-VEC with very low MOI values (0.00005 to 0.0005) (Supplementary Figures S9, S10 and S12). This observation might be explained by phenomena such as the induction of tumor cell senescence (going along with a tumor cell flattening), as the cell index also increases due to the enlargement of the tumor cells. One possible mechanism may reside in therapy-induced downregulation of Myc expression, which could be triggered by small molecule compounds and may reactivate cellular senescence programs in tumors in an oncogene-dependent manner [45]. 

Although our preliminary trials with immunoblot assays indicate that Myc could be strongly downregulated by dual combination treatment, this needs to be investigated much closer in future follow-up projects. For this reason, only speculations about possible explanations can be made at this stage, which will provide new starting points. While monotherapy with T-VEC increased the expression of Myc at 24 h, it decreased moderately at 48 hpi and abruptly at 72 hpi. In contrast, a dual combination with vorinostat, panobinostat or fimepinostat led to an earlier and more pronounced downregulation of Myc, which could indicate a mechanistic reason. Beyond that, T-VEC (like its parental wild-type HSV-1 virus) can alter signaling pathways and regulatory networks in cancer cells, which could affect the expression of Myc: HSV-1 is known to use viral proteins such as virion protein 16 (VP16) or infected cell protein 0 (ICP0) for its replication, which are associated with general transcription factors and chromatin-modifying coactivators, thus potentially influencing Myc promoters and modulating their activity for this purpose [46,47,48,49]. Another possibility could be that both the PI3K/Akt signaling pathway and the mitogen-activated protein (MAP) kinase pathway can be upregulated during HSV-1 infection, which could temporarily phosphorylate and inactivate repressors of Myc [50,51,52,53]. 

Monotherapy with HDACi compounds vorinostat, panobinostat and the dual PI3K/HDACi fimepinostat showed no notable changes in Myc expression in the first 24 h, compared to untreated HCC2429 cells. It is conceivable that HDACi can cause a reversible instability of Myc and a reactivation of epigenetically silenced genes only a few hours after application, as reported by previous studies in Myc-driven cancers [35,40], which could later be reversed and lead to basal Myc expression rates. Another effect of HDACi is the ability to induce apoptosis by suppressing the anti-apoptotic protein Bcl-2 [35,54] or the activation of the pro-apoptotic protein Bim by targeting the Rb/E2F pathway [55]. The Rb protein is a well-known key tumor suppressor, which controls the transition from the G1 phase to the S phase in the cell cycle by complex formation with E2F1 (E2F1-Rb) in an unphosphorylated state so that E2F1 cannot mediate the transcription of proteins required for DNA synthesis and the cell cycle [56,57]. Myc induces E2F1, and in turn, E2F1 can induce Myc [58], so it could be possible that this positive feedback loop is also involved in NC tumor cells. Interestingly, a hypophosphorylation of Rb and an accumulation of E2F1-Rb complex was also observed during HSV-1 infection of tumor cells, so that an initiated cell cycle can be blocked afterward [59]. Furthermore, it has been found that combination treatment by oncolytic HSV-1 with the HDACi compound trichostatin A can promote both replication and G1 arrest in oral squamous cell carcinoma [60].

In conclusion, we believe that dual combination therapy with T-VEC and SMI compounds may slow down cell cycle progress and lead to G1 arrest in NC cells, as one possible way to suppress Myc. In this process, the CDK4/6i palbociclib could be supportive by modulation of Rb and causing cell cycle arrest. P300/CBP inhibitors, such as GNE-781, may also be beneficial by co-regulating the cell cycle along this pathway. In addition, enhanced oncolysis by dual combination therapy could also be part of the downregulation of Myc expression, as the loading control bands are still clearly visible, albeit slightly diminished (Figure 5). However, further experiments and many more investigations are needed to confirm this first data set and to transfer it to other NC cancer cell lines, including those that do not carry BRD4 as a fusion partner, which may uncover additional co-actors and novel therapeutic targets. Furthermore, a genetically engineered mouse model (GEMM) of NC, involving the BRD4-NUT fusion gene, has very recently been developed for the first time [61]. With this important contribution, previous preclinical studies could be transferred in vivo as well to evaluate further mechanistic results, but access is currently limited. Nevertheless, it still remains possible to test our combinatorial approaches in patient-derived NC tumor cells or thereby establish organoid models and thus achieving improvements in therapeutic application.

## 5. Conclusions

Our promising in vitro results suggest that combinatorial approaches using the oncolytic virus T-VEC and SMIs (such as vorinostat, panobinostat, fimepinostat, GNE-781, or palbociclib) have great potential for future multimodal treatment approaches and may provide better success for the treatment of this highly challenging NC tumor type.

## Data Availability

The data presented in this study are available within this article and its Supplementary Data Files.

## Supplementary Materials

The following supporting information can be downloaded at: https://www.mdpi.com/article/10.3390/v16050775/s1, Figure S1: Viability of human NC cell line HCC2429 after monotherapeutic treatment with five small molecule inhibitors (SMIs); Figure S2: Viability of human NC cell line 143100 after monotherapeutic treatment with five small molecule inhibitors (SMIs); Figure S3: Viability of human NC cell line 10-15 after monotherapeutic treatment with five small molecule inhibitors (SMIs); Figure S4: Viability of human NC cell line 690100 after monotherapeutic treatment with five small molecule inhibitors (SMIs); Figure S5: Viability of human NC cell line 14169 after monotherapeutic treatment with five small molecule inhibitors (SMIs); Figure S6: Viability of human NC cell lines after monotherapeutic treatment with T-VEC; Figure S7: Viability of human NC cell line 690100 after dual combination treatment with T-VEC and small molecule inhibitors (SMIs); Figure S8: Viability of human NC cell line 14169 after dual combination treatment with T-VEC and small molecule inhibitors (SMIs); Figure S9: Real-time analysis of 143100 NC tumor cells under treatment with T-VEC alone or under dual combination therapies with the small molecule inhibitors (SMIs); Figure S10: Real-time analysis of 10-15 NC tumor cells under treatment with T-VEC alone or under dual combination therapies with the small molecule inhibitors (SMIs); Figure S11: Real-time analysis of 690100 NC tumor cells under treatment with T-VEC alone or under dual combination therapies with the small molecule inhibitors (SMIs); Figure S12: Real-time analysis of 14169 NC tumor cells under treatment with T-VEC alone or under dual combination therapies with the small molecule inhibitors (SMIs); Figure S13: Raw immunoblot data for the detection of c-Myc (Myc) after treatment of HCC2429 cells for 24 h with T-VEC and small molecule inhibitors (SMIs) alone or in dual combination; Figure S14: Raw immunoblot data for the detection of c-Myc (Myc) protein after treatment of HCC2429 cells for 48 h with T-VEC and small molecule inhibitors (SMIs) alone or in dual combination; Figure S15: Raw immunoblot data for the detection of c-Myc (Myc) protein after treatment of HCC2429 cells for 72 h with T-VEC and small molecule inhibitors (SMIs) alone or in dual combination.
