# Supplementary material for: Multimodal Therapy Approaches for NUT Carcinoma by Dual Combination of Oncolytic Virus Talimogene Laherparepvec with Small Molecule Inhibitors
Source: Viruses. 2024 May 14;16(5):775. doi: 10.3390/v16050775 (PMC11125747; doi:10.3390/v16050775)
Supplement: Supplementary file 1 [file viruses-16-00775-s001.zip › FigureS10.pdf]

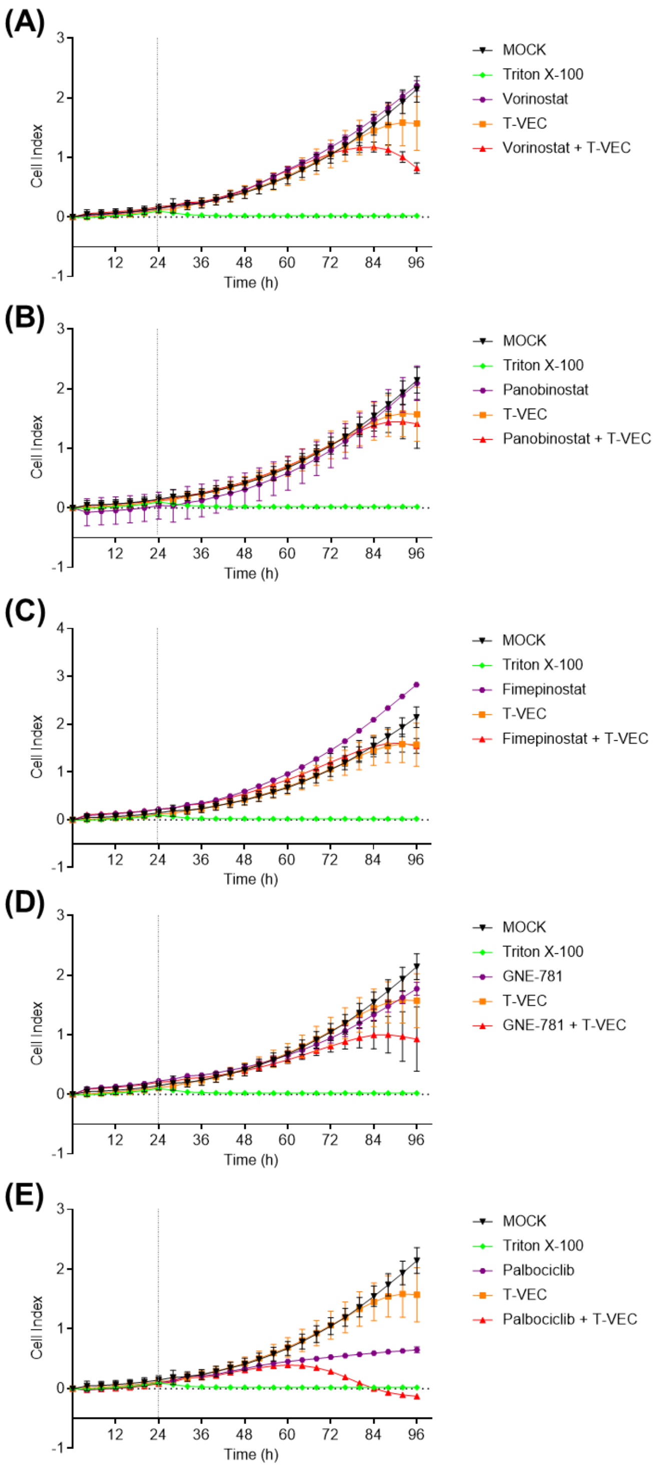

Figure S10. Real-time analysis of 10-15 NC tumor cells under treatment with T-VEC alone or under dual combination therapies with the small molecule inhibitors (SMIs). 10-15 tumor cells were treated 24 h after seeding with T-VEC (MOI 0.0005) or with the SMI at the indicated concentration alone (vorinostat (250 nM) (**A**), panobinostat (2.5 nM) (**B**), fimepinostat (0.5 nM) (**C**), GNE-781 (10 nM) (**D**), palbociclib (25 nM) (**E**)) or in combination. Triton X-100 0.1 % was added as negative control, resulting in maximal tumor cell lysis. Real-time cell proliferation was monitored using the xCELLigence® RTCA SP system 1.2.1. Cell index on y-axis is defined by measured electrode impedance. Vertical dashed lines mark the point of infection. MOCK, Triton X-100 and T-VEC curves are the same in each graph. Mean ± SD of one representative experiment at least performed in quadruplicates is shown.
