# Supplementary material for: Multimodal Therapy Approaches for NUT Carcinoma by Dual Combination of Oncolytic Virus Talimogene Laherparepvec with Small Molecule Inhibitors
Source: Viruses. 2024 May 14;16(5):775. doi: 10.3390/v16050775 (PMC11125747; doi:10.3390/v16050775)
Supplement: Supplementary file 1 [file viruses-16-00775-s001.zip › FigureS15.pdf]

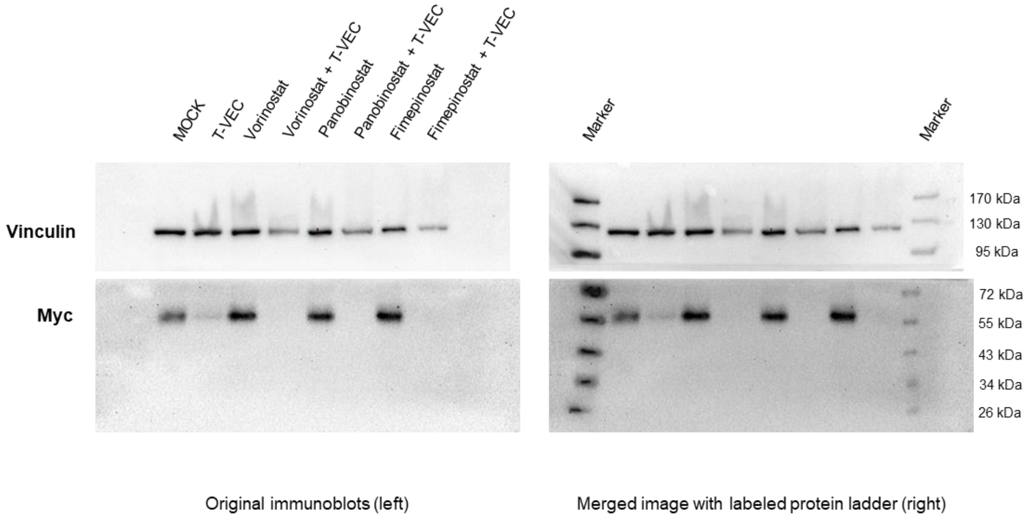

Figure S15. Raw immunoblot data for the detection of c-Myc (Myc) protein after treatment of HCC2429 cells for 72 h with T-VEC and small molecule inhibitors (SMIs) alone or in dual combination: HCC2429 cells were treated with T-VEC (MOI 0.001), vorinostat (500 nM), panobinostat (2.5 nM), fimepinostat (0.5 nM) alone or T-VEC in dual combination with a distinct SMI or remained untreated (MOCK). Anti-Vinculin antibody was used to provide loading control. All experimental samples and controls used are run on the same blot. The original images are displayed on the left-hand side, the merged image with labeled protein ladder (marker) and information on protein molecular weight in kilodaltons (kDa) is shown on the right-hand side. Cropped blots are presented in Figure 5.
