# Supplementary material for: Multimodal Therapy Approaches for NUT Carcinoma by Dual Combination of Oncolytic Virus Talimogene Laherparepvec with Small Molecule Inhibitors
Source: Viruses. 2024 May 14;16(5):775. doi: 10.3390/v16050775 (PMC11125747; doi:10.3390/v16050775)
Supplement: Supplementary file 1 [file viruses-16-00775-s001.zip › FigureS4.pdf]

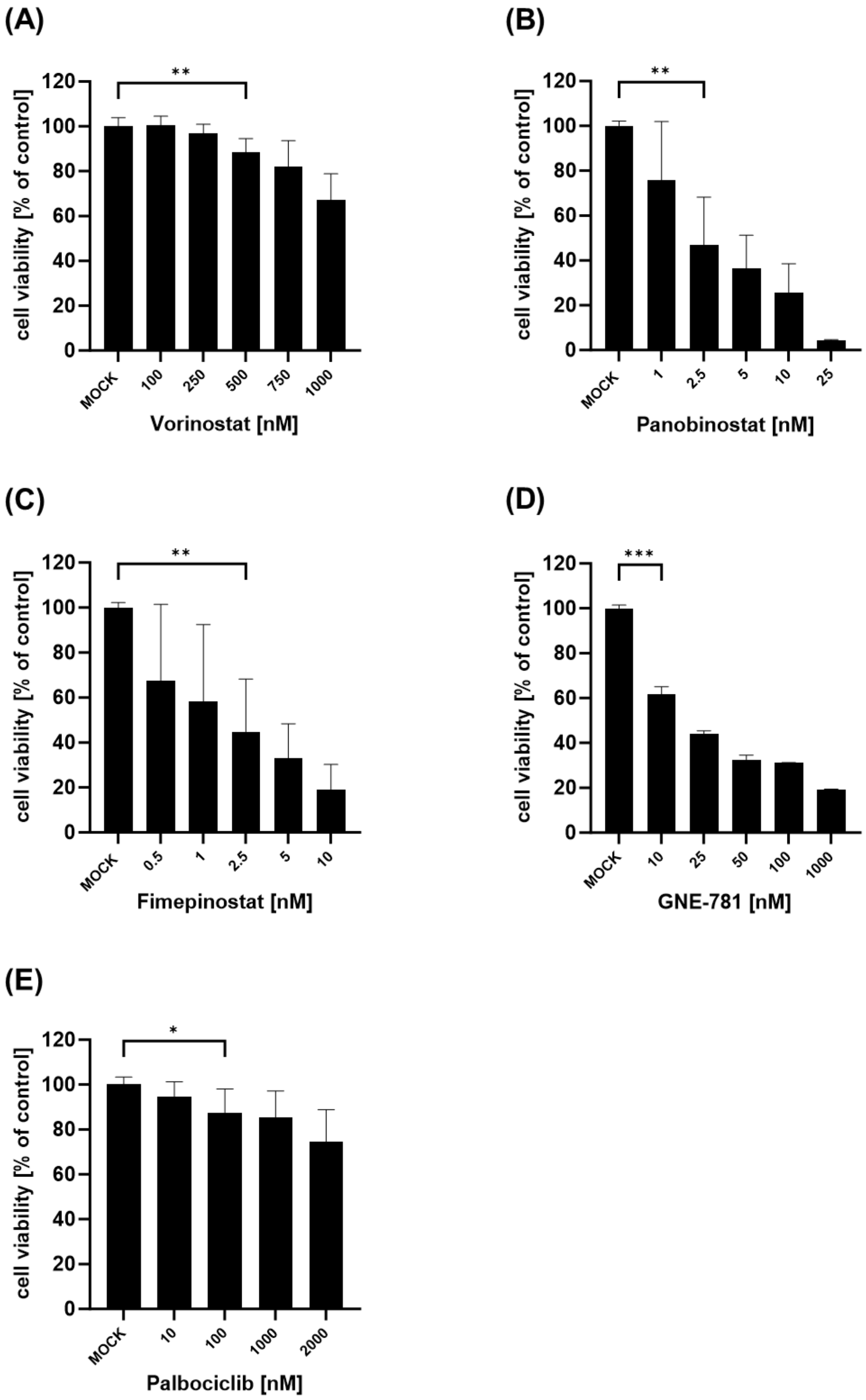

Figure S4. Viability of human NC cell line 690100 after monotherapeutic treatment with five small molecule inhibitors (SMIs): Tumor cells were mono-treated with vorinostat (**A**), panobinostat (**B**), fimepinostat (**C**), GNE-781 (**D**), palbociclib (**E**) at ascending concentrations or remained untreated (MOCK). The remaining NC tumor cells were determined at 96 h after treatment by SRB viability assays. The anti-tumor effects mediated by distinct SMI are calculated relative to MOCK controls. The mean ± SD of at least two independent experiments performed in triplicates is shown, except for GNE-781 with one performed experiment. Displayed significance levels refer to the lowest concentration that caused a significant result, compared to MOCK. * $p < 0.05$, ** $p < 0.01$, *** $p < 0.001$.
