# Supplementary material for: Multimodal Therapy Approaches for NUT Carcinoma by Dual Combination of Oncolytic Virus Talimogene Laherparepvec with Small Molecule Inhibitors
Source: Viruses. 2024 May 14;16(5):775. doi: 10.3390/v16050775 (PMC11125747; doi:10.3390/v16050775)
Supplement: Supplementary file 1 [file viruses-16-00775-s001.zip › FigureS6.pdf]

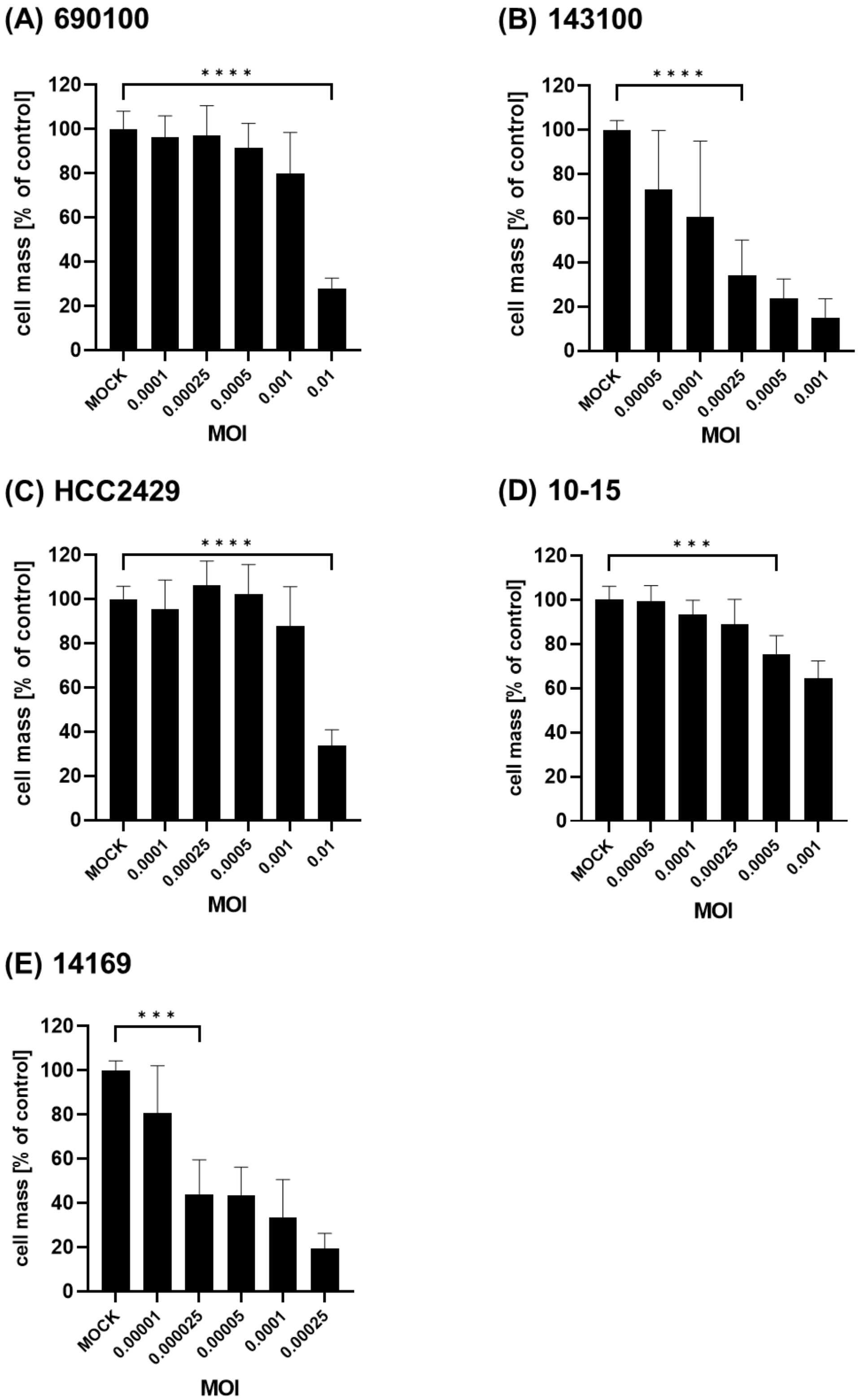

Figure S6. Viability of human NC cell lines after monotherapeutic treatment with T-VEC: NC tumor cells 690100 (**A**), 143100 (**B**), HCC2429 (**C**), 10-15 (**D**), 14169 (**E**) were infected with T-VEC at different multiplicities of infection (MOI) or remained uninfected (MOCK). Remaining NC tumor cells were determined at 72 h post-infection (hpi) by SRB viability assay. T-VEC-mediated oncolysis was calculated relatively to MOCK control. The mean ± SD of at least two independent experiments performed in quadruplicates is shown. Displayed significance levels refer to the lowest MOIs that caused a significant result, compared to MOCK. *** $p < 0.001$, **** $p < 0.0001$.
