# Supplementary material for: Multimodal Therapy Approaches for NUT Carcinoma by Dual Combination of Oncolytic Virus Talimogene Laherparepvec with Small Molecule Inhibitors
Source: Viruses. 2024 May 14;16(5):775. doi: 10.3390/v16050775 (PMC11125747; doi:10.3390/v16050775)
Supplement: Supplementary file 1 [file viruses-16-00775-s001.zip › FigureS7.pdf]

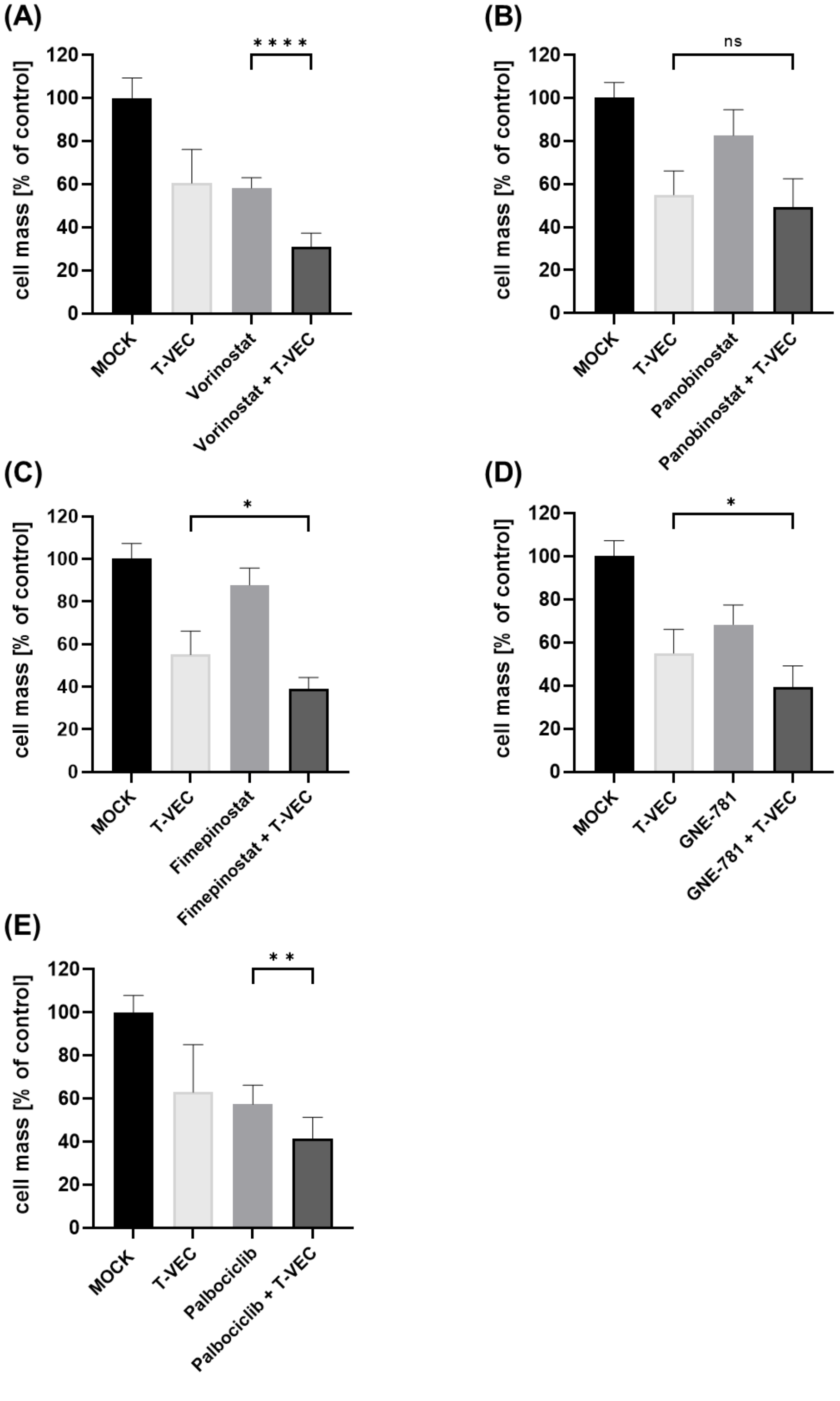

Figure S7. Viability of human NC cell line 690100 after dual combination treatment with T-VEC and small molecule inhibitors (SMIs): 690100 cells were infected with T-VEC at a multiplicity of infection (MOI) of 0.001 and treated with the SMIs vorinostat (1 µM) (**A**), panobinostat (1 nM) (**B**), fimepinostat (0.5 nM) (**C**), GNE-781 (10 nM) (**D**), palbociclib (3 µM) (**E**) in dual combination or alone, or remained untreated (MOCK). The remaining NC tumor cells were determined at 72 h post-infection (hpi) by SRB viability assays. The mean ± SD of at least two independent experiments performed in triplicates is shown. Reported significances refer to dual combination compared to the respective monotherapy with the lowest remaining cell viability. * $p < 0.05$; ** $p < 0.01$, **** $p < 0.0001$, n.s. not significant.
